# The *FTO* A/T Polymorphism and Elite Athletic Performance: A Study Involving Three Groups of European Athletes

**DOI:** 10.1371/journal.pone.0060570

**Published:** 2013-04-03

**Authors:** Nir Eynon, Emiliya S. Nasibulina, Lauren K. Banting, Pawel Cieszczyk, Agnieszka Maciejewska-Karlowska, Marek Sawczuk, Elvira A. Bondareva, Roza R. Shagimardanova, Maytal Raz, Yael Sharon, Alun G. Williams, Ildus I. Ahmetov, Alejandro Lucia, Ruth Birk

**Affiliations:** 1 School of Sports and Exercise Sciences, Victoria University, Melbourne, Australia; 2 Institute of Sport, Exercise and Active Living (ISEAL), Victoria University, Melbourne, Australia; 3 Laboratory of Molecular Genetics, Kazan State Medical University, Kazan, Russia; 4 Faculty of Tourism and Recreation, Academy of Physical Education and Sport, Gdansk, Poland; 5 Faculty of Physical Culture and Heath Promotion, University of Szczecin, Szczecin, Poland; 6 Institute and Museum of Anthropology, Moscow State University, Moscow, Russia; 7 Faculty of Health Sciences, Department of Nutrition, Ariel University Center, Ariel, Israel; 8 Centre for Genomics Research into Exercise, Performance and Health, Manchester Metropolitan University, Manchester, United Kingdom; 9 Sport Technology Education Research Laboratory, Volga Region State Academy of Physical Culture, Sport and Tourism, Kazan, Russia; 10 School of Doctorate Studies and Research, European University of Madrid, Madrid, Spain; University of Bath, United Kingdom

## Abstract

**Objective:**

The *FTO* A/T polymorphism (rs9939609) is a strong candidate to influence obesity-related traits. Elite athletes from many different sporting disciplines are characterized by low body fat. Therefore, the aim of this study was to assess whether athletic status is associated with the *FTO* A/T polymorphism.

**Subjects and Methods:**

A large cohort of European Caucasians from Poland, Russia and Spain were tested to examine the association between *FTO* A/T polymorphism (rs9939609) and athletic status. A total of 551 athletes were divided by type of sport (endurance athletes, n = 266 vs. sprint/power athletes, n = 285) as well as by level of competition (elite-level vs. national-level). The control group consisted of 1,416 ethnically-matched, non-athletic participants, all Europeans. Multinomial logistic regression analyses were conducted to assess the association between *FTO* A/T genotypes and athletic status/competition level.

**Results:**

There were no significantly greater/lesser odds of harbouring any type of genotype when comparing across athletic status (endurance athletes, sprint/power athletes or control participants). These effects were observed after controlling for sex and nationality. Furthermore, no significantly greater/lesser odds ratios were observed for any of the genotypes in respect to the level of competition (elite-level vs. national-level).

**Conclusion:**

The *FTO* A/T polymorphism is not associated with elite athletic status in the largest group of elite athletes studied to date. Large collaborations and data sharing between researchers, as presented here, are strongly recommended to enhance the research in the field of exercise genomics.

## Introduction

There is emerging evidence that elite athletes or former elite athletes are predisposed towards longer life expectancy, and lower risk of chronic diseases such as obesity and type 2 diabetes, than matched sedentary controls [1]–[3]. A twenty-year follow-up on former elite athletes revealed that several risk factors (smoking, diabetes, obesity) were never present among the athletes, and the prevalence of other risk factors remained low after twenty years [3].

Genetic factors may contribute to the low predisposition of elite athletes to the aforementioned disease conditions. Specifically, the A/T polymorphism (rs9939609) in the fat mass and obesity associated (*FTO*) gene (which codes for the protein alpha-ketoglutarate-dependent dioxygenase FTO, also known as fat mass and obesity-associated protein) is a strong candidate to explain how disease modifier polymorphisms may contribute to lower risk for obesity among trained individuals [4]. The *FTO* A/T polymorphism has initially been identified as a risk factor for obesity by two independent genome-wide association studies (GWAS) [5], [6]. It has been shown that adults who are homozygous for the A-allele weigh on average 1.5 to 3 kg more than those homozygous for the T allele. This finding has now been replicated in multiples obese cohorts [7].

Exercise may attenuate the association between *FTO* A/T polymorphism and obesity related-traits. The association between *FTO* A/T polymorphism and body mass index (BMI) is significantly weaker in individuals with higher exercise levels [8]. This phenomenon has been confirmed in Caucasian and African-American cohorts [9], [10]. A recent meta-analysis data, that was calculated from 45 studies of adults (n = 218,166) and 9 studies of children and adolescents (n = 19,268) has shown that the A-allele increased the risk of obesity 30% less in the physically active group than in their inactive peers [4]. Keeping in mind that elite athletes represent the end point of the human physical activity levels, the *FTO* A/T polymorphism might be a novel target to influence elite athletic status.

Therefore, the aim of the present study was to compare the frequency distribution of the *FTO* A/T polymorphism (rs9939609) between elite endurance athletes, elite sprint/power athletes, and ethnically-matched, non-athletic control participants in a large group of Europeans (including Spanish, Polish and Russian cohorts). We also examined the association of the *FTO* A/T polymorphism with respect to the level of achievement of the athletes (‘elite-level’ and ‘national-level’), in both endurance and sprint/power athletes. We hypothesized that the frequency of the A-allele or the AA genotype will be lower among elite athletes compared with control participants.

## Materials and Methods

The study was conducted according to the Declaration of Helsinki. Written informed consent was obtained from all participants, and the study was approved by the ethics committees of *Universidad Europea de Madrid*, Spain, the *Pomeranian Medical University*, Poland, and the *Kazan State Medical University*, Russia.

### Participants

A total of 551 athletes (266 endurance athletes and 285 power athletes) and 1416 control participants, from Poland, Russia and Spain, volunteered to participate in this study. All participants were unrelated European men (76%) or women (23%), and all of European descent (as self-reported) for ≥3 generations. The sample included elite athletes (57%) who had competed at an international level (European or World championships, or Olympic Games) and national-level athletes (43%) who participated in their chosen sport at a national level only. The competition level differentiation was made according to the athletes' best individual performances. The athletes were only included if they had never tested positive in anti-doping controls. Control participants were required to be free of any diagnosed cardiorespiratory disease and not participating regularly in any competitive or structured sport or physical activity (i.e. performing less than 3 sessions per week of strenuous exercise such as running, swimming, bicycling or weight lifting).

#### Spanish cohort

The Spanish cohort (n = 192) were all male and included 81 elite athletes (mean ± SD mass = 62.0±6.3 kg) and 60 control participants (71.9±8.3 kg):

- 32 elite sprint/power athletes (mean age = 26±3 yr). Thirteen track and field athletes were Olympians during the period 2000–2008.
- 49 elite endurance athletes (27±4 yr). This sample included 49 elite endurance runners (the top Spanish runners during the 1999–2009 periods, i.e. mainly 5000 m to marathon specialists, virtually all of them Olympians).
- 60 healthy, non-athletic control participants (20±2 yr). All were undergraduate students from the same university (*Universidad Europea de Madrid*, Spain).

#### Polish cohort

The Polish cohort (n = 844), were all male and included 214 athletes (mean ± SD mass = 71.3±6.2 kg) and 630 control participants (79.2±6.1 kg). Of the athletes, 132 were classified as elite and 82 were national-level athletes:

- 101 power athletes (28±8 yr). This group included weightlifters (n = 42), sprinters (≤400 m, n = 33), and track and field jumpers (n = 26). This group included 63 (62%) elite athletes.
- 113 endurance athletes (26±6 yr). This group included rowers (n = 53), endurance road cyclists (n = 14), 5,000 m runners (n = 12), marathon runners (n = 12), 800–1,500 m swimmers (n = 11), 15–50 km cross-country skiers (n = 7), and triathletes (n = 4). This group included 69 (61%) elite athletes.
- 630 healthy, non-athletic control participants (21±2 yr). All the control participants were students of the University of Szczecin.

#### Russian cohort

The Russian cohort (men and women, n = 982) included 256 athletes (187 men, 69 women; 70.3±16.8 kg) and 726 control participants (328 men and 398 women; 61.2±12.2 kg). Of the athletes 105 were classified as elite and 151 were classified as national-level athletes:

- 152 power athletes (24±8 yr). This group included: 100–200 m sprinters (n = 18), track and field jumpers (n = 47), short distance speed skaters (500–1000 m; n = 9), 50–100 m swimmers (n = 13), and weight lifters (n = 65). The group included 71 (47%) elite athletes.
- 104 endurance athletes (20±2 yr). This group included rowers (n = 36), long distance runners (5000 m; n = 27), road cyclists (n = 12), long distance speed skaters (5–10 km; n = 7), skiers (n = 15) and long distance swimmers (800–1500 m; n = 7).The group included 34 (33%) elite athletes.
- 726 healthy, non-athletic control participants (21±5 yr). All the control participants were citizens of Moscow and Kazan.

### Genotyping

We followed recent recommendations for genotype-phenotype association studies provided by Chanock et al. [11], Attia et al. [12] and the latest ‘Strengthening the Reporting of Genetic Association studies’ (STREGA) group report [13].

#### Spanish cohort

Genomic DNA was isolated from buccal epithelium or peripheral blood during the years 2004–2008 and genotyping was performed during 2012 in the Genetics Laboratory of *Ariel University Centre, Israel*. Polymerase chain reaction (PCR) was performed in order to amplify the sequence containing the mutation. A fragment of 105 bp was amplified with the following primers: *FTO*- F 5′- GGT TCC TTGCGA CTG CTG TGA AAT T '3 and *FTO*-R 5' GCT TTT ATGCTC TCC CAC TC '3. The PCR conditions were as follows: initial denaturing at 95°C 5 min; 35 cycles at 95°C 30 s, 60°C 30 s, 72°C 30 s and a final extension at 72°C 10 min. *FTO* genotypes were established by enzymatic digestion of amplicons with *ApoI* and by allelic discrimination assay on a Real-Time Polymerase Chain Reaction (PCR) instrument (Stratagene Mx3000D) with Taqman® probes (Genotyping ToughMix®). Following recent recommendations [11], we replicated the genotype results of the Spanish cohort (in 40% of samples) using a different method, i.e. direct sequencing. The results from the two different methods were in 100% agreement.

#### Polish cohort

Genomic DNA was isolated from buccal epithelium using GenElute Mammalian Genomic DNA Miniprep Kit (Sigma, Germany) during the years 2008–2010, according to the producer protocol. All samples were genotyped during 2012, in the *Pomeranian Medical University* using an allelic discrimination assay on a Rotor-Gene Real-Time Polymerase Chain Reaction (PCR) instrument (Corbett, Australia) with Taqman® probes. For the discrimination of *FTO* A and T alleles (rs9939609), a TaqMan® Pre-Designed SNP Genotyping Assay was used (Applied Biosystems, USA) (assay ID: C__30090620_10), including primers and fluorescently labelled (FAM and VIC) MGB™ probes for the detection of both alleles.

#### Russian cohort

Genomic DNA was isolated from epithelial mouth cells using a DNK-sorb-A sorbent kit according to the manufacturer's instruction (Central Research Institute of Epidemiology, Moscow, Russia). Genotyping for the *FTO* gene polymorphism was performed during 2012, at the *Laboratory of Molecular Genetics, Kazan State Medical University* by PCR on a multicanal amplificator Tercyk (DNA Technology, Moscow, Russia) and restriction enzyme digestion [14].

Following recent recommendations [11], we replicated the genotype results of a subset of samples (i.e. 40% of samples of the Russian cohort) using a different method, i.e. by MALDI-TOF mass spectrometry [15].

### Statistical analysis

Chi squared tests were used to test for the presence of Hardy-Weinberg equilibrium (HWE). Genotype and allele frequencies were compared according to athletic status (i.e. control participants, endurance, or sprint/power athlete) using Fisher's exact test. Multinomial logistic regression analyses were conducted to assess the association between genotype and athletic status/competition level. In each case, gender and nationality were controlled for; and analyses were made comparing AA (reference group) vs. AT; AA vs. TT (co-dominant effect); AA vs. TT and TA combined (dominant effect); AA and TA combined (reference group) vs. TT (recessive effect). Significance was accepted when p≤0.05.

## Results

Replication analysis with a different genotyping method yielded 100% agreement. There were no significant differences in age between cohorts. This added to the homogeneity of the study population and allowed us to pool the three cohorts to examine the association between physical performance level and *FTO* A/T polymorphism.

Table 1 shows the genotype and allele frequency distributions amongst all participants according to their nationality. Genotype distributions of all control and athletic groups met HWE (all *p*>0.1). In the Russian sample, no differences were observed in the proportion of men and women participating in endurance and power sports (*p*>0.05) and genotype and allele frequencies were similar according to gender (*p*>0.05; data not shown). For these reasons, gender was considered as a covariate only in further analyses (see below).

**Table 1:** *FTO* A/T polymorphism genotype and allele frequencies amongst all participants according to their nationality.

	Spanish cohort			Polish cohort			Russian cohort		
	Control	Endurance	Power	Control	Endurance	Power	Control	Endurance	Power
All	60	49	32	630	113	101	726	104	152
AA	5 (8.3)	5 (10.2)	4 (12.5)	119 (18.9)	13 (11.5)	19 (18.8)	111 (15.3)	17 (16.3)	27 (17.8)
AT	7 (11.7)	14 (28.6)	7 (21.8)	318 (50.5)	65 (57.5)	52 (51.5)	324 (44.6)	54 (51.9)	68 (44.7)
TT	48 (80)	30 (61.2)	21 (65.6)	193 (30.6)	35 (31.0)	30 (29.7)	291 (40.1)	33 (31.7)	57 (37.5)
MAF	0.141	0.245	0.234	0.441	0.403	0.446	0.376	0.423	0.401
HWE-*P* value	.001	0.282	0.086	0.838	0.115	0.914	.001	0.810	0.696

*Abbreviations*: HWE, Hardy-Weinberg equilibrium; MAF, minor allele frequency.

Table 2 shows the association between genotype and athletic status for all participants. There were no significantly greater/lesser odds of harbouring any type of genotype when comparing the power, endurance and control groups. Likewise, no differences were observed when comparing all athletes to the control group. These effects were observed after controlling for the effects of gender and nationality.

**Table 2:** The association between *FTO* A/T genotypes and athletic status in three cohorts of European participants.

	Power vs. Control			Endurance vs. Power			Endurance vs Control			All athletes vs Control		
	OR	CI	*p*	OR	CI	*p*	OR	CI	*p*	OR	CI	*p*
AA (ref)	1			1			1			1		
AT	0.95	(0.66–1.36)	0.782	1.48	(0.89–2.45)	0.129	1.46	(0.97–2.18)	0.070	1.15	(0.86–1.54)	0.349
TT	1.01	(0.70–1.46)	0.968	1.16	(0.69–1.96)	0.569	1.30	(0.85–1.98)	0.229	1.11	(0.82–1.50)	0.484
AT-TT (AA ref)	0.98	(0.70–1.37)	0.892	1.33	(0.82–2.14)	0.238	1.39	(0.94–2.05)	0.097	1.14	(0.86–1.49)	0.363
TT (AA-AT ref)	1.05	(0.80–1.36)	0.742	0.87	(0.61–1.24)	0.426	0.98	(0.74–1.29)	0.858	1.00	(0.82–1.24)	0.969

*Note*. OR: Odds ratio; CI: Confidence intervals; *p*: 2-tailed *p* value with significance assumed at *p*<0.05.

Table 3 shows the association between genotype and competition level (elite vs. national level) for the endurance and power athletes from all countries. No significantly greater/lesser odds ratios were observed for any of the genotypes in either competition level. As above, gender and nationality were controlled for in the regression analyses.

**Table 3:** The association between *FTO* A/T genotypes and athletic status according to level of competition (elite compared to national level), in three cohorts of European participants.

	Endurance			Power		
	OR	CI	*p*	OR	CI	*p*
AA (ref)	1			1		
AT	0.79	(0.22–2.87)	0.719	1.61	(0.79–3.27)	0.187
TT	2.08	(0.83–5.23)	0.116	1.93	(0.93–4.03)	0.079
AT-TT (AA ref)	2.14	(0.93–4.96)	0.076	1.75	(0.90–3.39)	0.099
TT (AA-AT ref)	1.13	(0.63–2.05)	0.682	1.37	(0.81–2.33)	0.241

*Note*. OR: Odds ratio; CI: Confidence intervals; *p*: 2-tailed *p* value with significance assumed at *p*<0.05.

**Figure 1:**
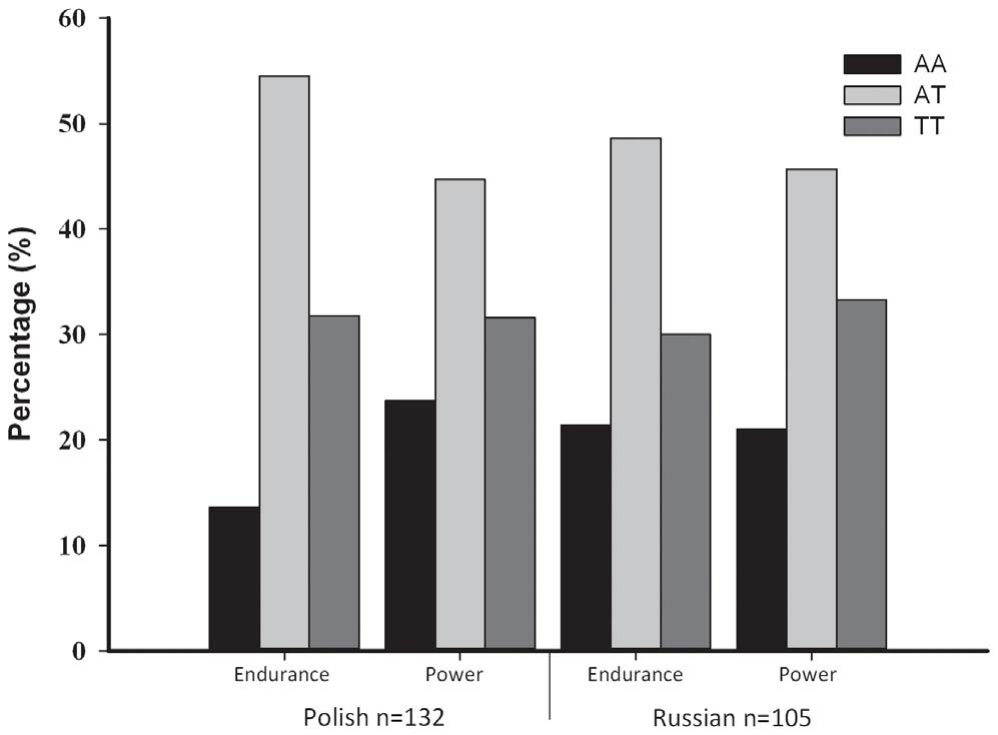
Genotype distributions in elite-level athletes according to nationality and athletic status.

Figure 1 shows the percentage of genotypes present in elite-level athletes according to nationality and athletic status. No significant genotype differences were observed between elite endurance athletes and elite power athletes across nationalities.

**Figure 2:**
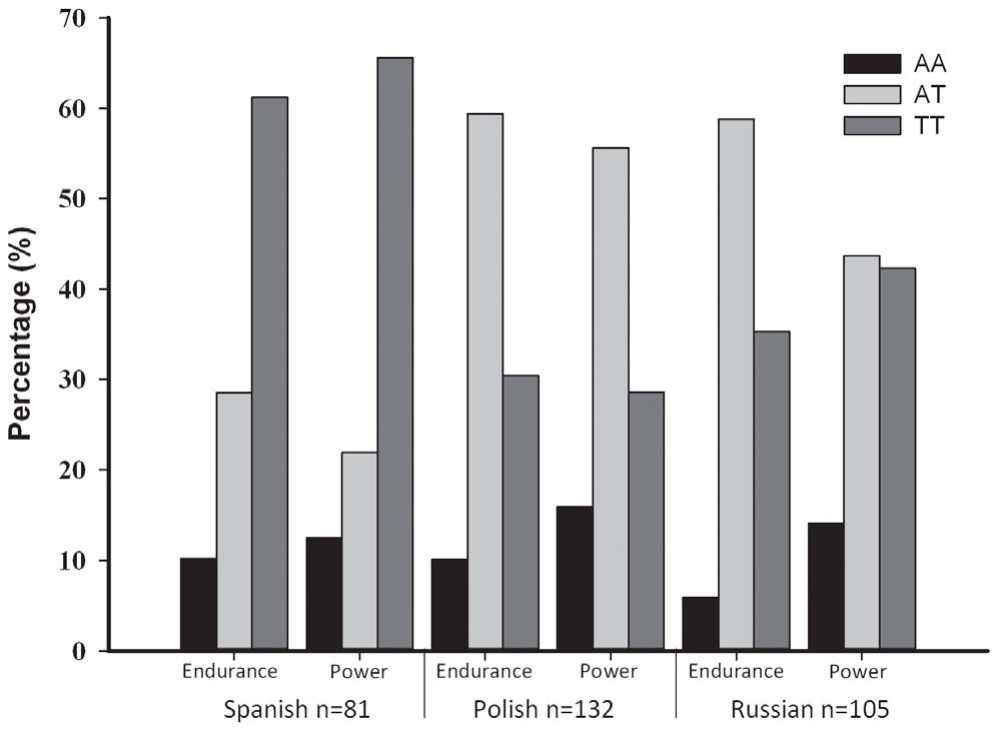
Genotype distributions in national-level athletes according to nationality and athletic status.

Figure 2 shows the percentage of genotypes present in national-level athletes according to nationality and athletic status. No significant genotype differences were observed between national-level endurance athletes and national-level power athletes across nationalities.

## Discussion

We have analysed, for the first time, the association between *FTO* A/T polymorphism (rs9939609) and elite athletic status in a large cohort of European athletes. No significant differences were observed in genotype/allele frequencies when comparing between the three groups (endurance athletes, sprint/power athletes, and non-athletic controls) or when comparing between the groups with respect to the level of performance (elite vs. national-level), suggesting that this polymorphic marker is not related to elite athletic performance.

In the present study we explored the association between the *FTO* A/T polymorphism and athletic performance in elite athletes from several cohorts. Taken individually, the results from our three cohorts are probably limited by relatively small sample size of the individual cohorts, and low statistical power. In an attempt to overcome the barrier of sample size, and the difficulty of gathering a homogenous cohort of elite athletes in each sporting discipline, we have recruited a total of 551 athletes (266 endurance athletes and 285 power/sprint athletes), all of European descent for ≥3 generations. Indeed, it has been estimated that testing a single variant using a case (athletes):control (non-athletes) design would require ∼250 cases to obtain a statistical power of 80% [16]. A sufficient sample size of elite athletes, together with following recent recommendations for association studies, are probably necessary to reach solid conclusions in the field of genes and elite performance [17].

Genetic variants such as the *FTO* A/T polymorphism studied here are associated with increased BMI and energy intake [9], [18], and are thus candidates to influence obesity and other disease-related phenotypes. Conversely, such variants may also influence elite athletic performance because body composition and BMI are well-characterised phenotypes in athletic populations that, to some extent at least, differentiate between athletes of different achievement levels, and between athletes and non-athletes. However, there are some complex interrelationships between increased/decreased BMI and both physical activity levels (i.e., energy expenditure) and energy intake, affected by interconnected metabolic processes [18], [19]. Indeed, physical activity was recently shown to attenuate the influence of *FTO* variants on obesity risk [4].

The duality of specific polymorphisms associated with both obesity and athletic performance has been well demonstrated. A good example is the peroxisome proliferator-activated receptor gamma coactivator1α (*PPARGC1A*) Gly482Ser polymorphism, in which the 482Ser allele is associated with increased risk of obesity and type 2 diabetes [20], whereas the ‘favourable’ Gly482 allele is associated with elite athletic performance [21]–[24]. Interestingly, the minor Ser482 allele is associated with risk of obesity in inactive individuals [20], [25] supporting the notion that genetic susceptibility to obesity is enhanced by physical inactivity. The link between *PPARGC1A* gene and fat oxidative metabolism suggest that this gene may influence athletic performance on one hand, and prevention of obesity on the other hand. Additional examples of polymorphisms that were found to be associated with both obesity and elite athletic performance are the *ADRB2* Arg16Gly (rs1042713) [26], [27], and the *ADRB3* Trp64Arg (rs4994) [28], [29].

The *FTO* A/T polymorphism (rs9939609) is located in the first intron of the *FTO* gene, which is expressed mainly in the brain, skeletal muscles and adipose tissue [30]. The mechanism by which it influences adiposity and attenuates physical activity is mostly unknown and probably multifaceted. Mechanistic research involving mice models demonstrated alternation in food intake in mice expressing several copies of the *FTO* gene, and significant reduction in adipose tissue and lean body mass [31]. A knockout mice model revealed that the *FTO* gene is functionally involved in energy homeostasis, mitochondrial coupling and substrate cycling by controlling energy expenditure [32]. The *FTO* A/T polymorphism was shown to affect energy efficiency potentially by influencing mitochondrial coupling in human type I (oxidative) muscle fibres [33], and *FTO* mRNA expression in human skeletal muscle correlates with whole-body substrate oxidation rates [34]. Thus, it could have been hypothesized that elite endurance performance in particular, which traditionally requires a high proportion of type I skeletal muscle fibres in the locomotory muscles and high mitochondrial coupling, would be influenced by the *FTO* A/T polymorphism.

To summarize, (i) the potential involvement of the *FTO* gene in energy metabolism and muscle function, (ii) the fact that other gene polymorphisms have been shown to be associated with both obesity and athletic performance (e.g. *PPARGC1A* Gly482Ser, *ADRB3* Trp64Arg, and the *ADRB2* Arg16Gly), (iii) the interaction between the *FTO* A/T polymorphism and physical activity levels, (iv) the low percentage of body fat characteristic of elite athletes excelling in both endurance and many sprint/power events (v) the lower risk of obesity in former elite athletes, and (vi) the suggestive role of the *FTO* gene in muscle performance, encouraged us to hypothesize that the *FTO* A/T polymorphism was associated with elite athletic status. However, no association was found between the *FTO* A/T polymorphism and athletic status in the largest group of elite athletes studied to date. Elite athletic status is a polygenic trait with several candidate gene variants (most of which likely remain unidentified) playing a certain role, either alone (which does not seem to be the case with the *FTO* A/T variation), or through complex, gene-gene and gene-environment interactions [35], [36]. Large collaborations and data sharing between researchers, as presented here, are strongly recommended to enhance the research in the field of exercise genomics.
